# Inhibition of nuclear factor-kappa B differentially affects thyroid cancer cell growth, apoptosis, and invasion

**DOI:** 10.1186/1476-4598-9-117

**Published:** 2010-05-21

**Authors:** Kevin T Bauerle, Rebecca E Schweppe, Bryan R Haugen

**Affiliations:** 1 Department of Medicine, Division of Endocrinology, Metabolism, and Diabetes, University of Colorado - Anschutz Medical Campus, Research Complex I, South Tower, Mail Stop 8106, 12801 East 17th Avenue, PO Box 6511, Aurora, CO 80045, USA; 2 Program in Cancer Biology, University of Colorado - Anschutz Medical Campus, Research Complex I, South Tower, Mail Stop 8104, 12801 East 17th Avenue, Aurora, CO 80045, USA; 3 University of Colorado Cancer Center, Aurora, CO 80045 USA

## Abstract

**Background:**

Nuclear factor-κB (NF-κB) is constitutively activated in many cancers and plays a key role in promoting cell proliferation, survival, and invasion. Our understanding of NF-κB signaling in thyroid cancer, however, is limited. In this study, we have investigated the role of NF-κB signaling in thyroid cancer cell proliferation, invasion, and apoptosis using selective genetic inhibition of NF-κB in advanced thyroid cancer cell lines.

**Results:**

Three pharmacologic inhibitors of NF-κB differentially inhibited growth in a panel of advanced thyroid cancer cell lines, suggesting that these NF-κB inhibitors may have off-target effects. We therefore used a selective genetic approach to inhibit NF-κB signaling by overexpression of a dominant-negative IκBα (mIκBα). These studies revealed decreased cell growth in only one of five thyroid cancer cell lines (8505C), which occurred through a block in the S-G2/M transition. Resistance to TNFα-induced apoptosis was observed in all cell lines, likely through an NF-κB-dependent mechanism. Inhibition of NF-κB by mIκBα sensitized a subset of cell lines to TNFα-induced apoptosis. Sensitive cell lines displayed sustained activation of the stress-activated protein kinase/c-Jun NH2-terminal kinase (SAPK/JNK) pathway, defining a potential mechanism of response. Finally, NF-κB inhibition by mIκBα expression differentially reduced thyroid cancer cell invasion in these thyroid cancer cell lines. Sensitive cell lines demonstrated approximately a two-fold decrease in invasion, which was associated with differential expression of MMP-13. MMP-9 was reduced by mIκBα expression in all cell lines tested.

**Conclusions:**

These data indicate that selective inhibition of NF-κB represents an attractive therapeutic target for the treatment of advanced thyroid. However, it is apparent that global regulation of thyroid cancer cell growth and invasion is not achieved by NF-κB signaling alone. Instead, our findings suggest that other important molecular processes play a critical role in defining the extent of NF-κB function within cancer cells.

## Background

Thyroid cancer is the most common endocrine malignancy [1]. Fortunately, a majority of patients are managed successfully with a combination of radioiodine and levothyroxine treatment following complete thyroidectomy. However, a subset of patients with advanced/dedifferentiated cancer have radioiodine-refractory disease with associated morbidity and mortality [2]. Given the high frequency of activating mutations in the mitogen-activated protein kinase (MAPK) pathway achieved by rearrangements associated with the RET tyrosine kinase and activating point mutations in RAS and BRAF [3], therapies targeting this pathway have been an area of active investigation [4]. Unfortunately, results from clinical studies regarding the overall efficacy of these therapies have been modest [5]. Clearly, there remains a need for a better understanding of the molecular events involved in thyroid cancer initiation and progression to aid in the identification of novel therapeutic targets.

The nuclear factor-κB (NF-κB) family of transcription factors is comprised of RelA (p65), RelB, c-REL, NF-κB1/p50, and NF-κB2/p52, each of which is characterized by a Rel homology domain, which facilitates DNA-binding, homo- or heterodimerization of NF-κB family members, and interaction with inhibitory IκB proteins. A role for NF-κB in oncogenic progression has been described in a number of lymphoid malignancies and carcinomas, including thyroid, ovarian, breast, and hepatocellular carcinomas [6]. Moreover, constitutive activation of NF-κB in tumors has been attributed to both excessive, chronic inflammation and activation by oncoproteins, as observed in hepatitis-induced hepatocellular carcinoma and melanoma, respectively [7,8]. NF-κB activation has also been implicated in acquired resistance to chemotherapy and radiation [9,10]. The end-product of NF-κB activation in cancer is believed to entail enhanced cell proliferation and invasion, as well as resistance to apoptosis induced by tumor surveillance mechanisms and various therapeutic modalities [10,11].

While the two primary modes of NF-κB activation are similar in that they culminate in NF-κB-dependent gene regulation through nuclear translocation of NF-κB dimers, the pathways are distinguished by the differential requirement of the trimeric IκB kinase (IKK) complex, which is composed of two kinase subunits, IKKα and IKKβ, and a regulatory, scaffolding subunit IKKγ. The classical pathway of activation requires phosphorylation of IκB proteins by the trimeric IKK complex, resulting in proteasome-dependent degradation of the inhibitory proteins and nuclear translocation of the classical p50/p65 heterodimer. The alternative pathway involves cleavage of the NF-κB2 precursor protein into the functional p52 subunit, which may then complex with RelB. This pathway is dependent on phosphorylation of the NF-κB2 precursor by IKKα dimers [12].

To date, several studies have employed the use of pharmacologic inhibitors of NF-κB to establish a role for NF-κB in thyroid cancer cell growth and invasion [13-17]. However, these results should be interpreted with caution given the potential for off-target effects of many of these drugs. In this report, we used a selective genetic inhibitor of NF-κB (mIκBα) in a panel of authenticated thyroid cancer cell lines [18]. We demonstrate that inhibition of NF-κB decreases thyroid cancer cell proliferation and invasion, while promoting TNFα-induced apoptosis. These findings are observed in only a subset of thyroid cancer cell lines and appear to be associated with distinct regulatory mechanisms.

## Results

### Inhibition of Thyroid Cancer Cell Growth by Pharmacologic Inhibition of NF-κB

Pharmacologic inhibitors of NF-κB have been widely used to investigate the functional consequences of constitutive NF-κB activation in cancer. Many of these inhibitors prevent phosphorylation and degradation of IκBα by blocking IKK complex activity [19]. We initially tested three NF-κB inhibitors, Bay 11-7082 (1 μM), IKK Inhibitor VII (1 μM), and CDDO-Me (0.25 μM), to investigate the role of NF-κB in thyroid cancer cell growth. The concentrations used in these experiments were based on studies using these compounds to document NF-κB-dependent effects on cell growth [20-22]. Each compound demonstrated inhibition of IKKβ activity by blocking TNFα-induced nuclear localization of p65 in a dose-dependent manner (data not shown).

**Figure 1:**
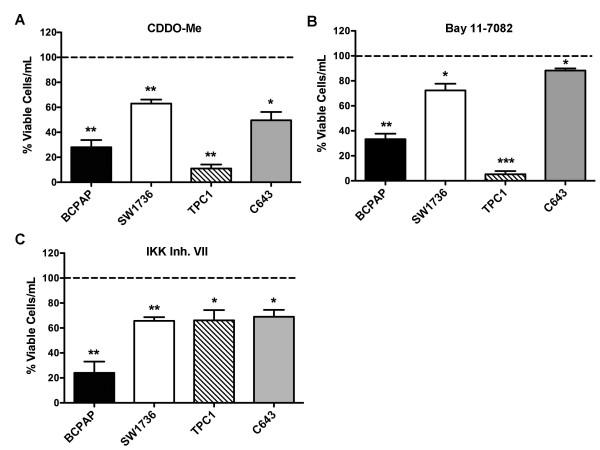
**Three Pharmacologic Inhibitors of NF-κB Differentially Decrease Growth of Thyroid Cancer Cell Lines**. BCPAP, SW1736, TPC1, and C643 cells were treated with vehicle (DMSO), (A) CDDO-Me (0.25 M), (B) Bay-11-7082 (1 M), or (C) IKK Inhibitor VII (1 M) for 5 days. Effect of treatment on proliferation and survival was determined by automated viable cell counting. Data is represented as percent viable cells/mL compared to vehicle control (normalized to 100%), which is denoted by the dashed line. Mean ± S.E.M. of 3 independent experiments performed in duplicate is reported. [p < 0.05 (*); p < 0.01 (**); p < 0.001 (***)].

A panel of papillary thyroid cancer (PTC) (BCPAP, TPC1) and anaplastic thyroid cancer (ATC) (SW1736, C643) cell lines were used. These cell lines harbour different activating mutations in the MAPK pathway, including the HRAS G13R mutation (C643), the BRAF V600E mutation (BCPAP, SW1736), and the RET/PTC1 rearrangement (TPC1) [18]. Treatment with CDDO-Me, Bay 11-7082, and IKK Inhibitor VII inhibited growth in all cell lines. Interestingly, the effects of the inhibitors on TPC1 (RET/PTC1) and C643 (HRAS) cells were quite variable, while the cells harbouring a BRAF V600E mutation (BCPAP and SW1736) displayed similar degrees of sensitivity to each of the inhibitors (Figure 1). The variable growth inhibition of these cell lines in response to treatment with three NF-κB inhibitors suggests that these inhibitors may exert their growth inhibitory effects through off-target mechanisms that are independent of NF-κB signaling.

### Inhibition of NF-κB by Adenoviral-mediated Overexpression of mIκBα

**Figure 2:**
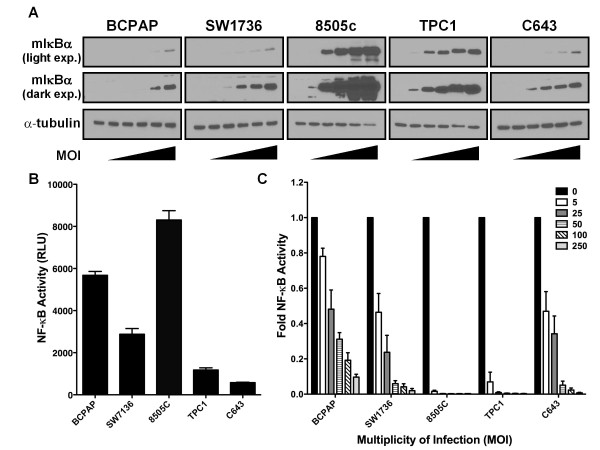
**Adenoviral-mediated Expression of mIκBα Blocks NF-κB Transcriptional Activity**. **(A) **BCPAP, SW1736, 8505C, TPC1, and C643 cells were transduced with Ad-mIκBα at an MOI of 0, 5, 25, 50, 100, and 250. After 48 hours, whole cell lysates were prepared, and expression levels of mIκBα were assessed by Western blot analysis. α-tubulin was used as a loading control. **(B) **Cells were transfected with an NF-κB-responsive luciferase reporter and actin-β-gal expression vector to control for transfection efficiency. Cell lysates were prepared 24 hours after transfection, after which luciferase and β-gal activities were assayed. Data is represented as NF-κB activity [luciferase activity/β-gal activity; relative light units (RLU)]. Mean ± S.E.M. of 3 independent experiments performed is reported. **(C) **Cells were transduced with Ad-mIκBα at an MOI of 0, 5, 25, 50, 100, and 250. After 24 hours, cells were then transfected with an NF-κB-responsive luciferase reporter and actin-β-gal expression vector to control for transfection efficiency. Cell lysates were prepared 24 hours after transfection, after which luciferase and β-gal activities were assayed. Data is represented as fold NF-κB activity where the basal activity (untransduced) of each cell line was normalized to 1. Mean ± S.E.M. of 3 independent experiments performed is reported.

Based on the variable growth inhibition by different pharmacologic inhibitors of NF-κB, a selective genetic approach to inhibit NF-κB signaling was used. Specifically, expression of a dominant-negative IκBα (mIκBα) [23,24], which is resistant to IKK-induced phosphorylation and proteasomal degradation, was carried out by adenoviral transduction [25]. This results in cytoplasmic sequestration and transcriptional inactivation of the NF-κB family of proteins. For these studies, we added the 8505C ATC cell line, which is characterized by the BRAF V600E mutation [18]. Adenoviral-mediated expression of mIκBα was assessed by Western blot analysis following transduction of thyroid cancer cell lines with a multiplicity of infection (MOI) ranging from 5-250 (Figure 2A). Expression levels of mIκBα varied substantially across the five cell lines tested, likely because of the efficiency of viral transduction. Indeed, a direct correlation between transduction efficiency, which was assessed by adenoviral-mediated expression of GFP, and mIκBα expression was observed (Figure 2A and data not shown).

The five thyroid cancer cell lines demonstrated different basal levels of NF-κB transcriptional activity as determined by an NF-κB-responsive luciferase reporter, with the BCPAP and 8505C cell lines exhibiting the highest levels of NF-κB activity (Figure 2B). Basal NF-κB activity did not correlate with tumor type (ATC vs. PTC). Figure 2C shows that NF-κB transcriptional activity could be inhibited by greater than 90% in each of the five cell lines. The degree of inhibition at a given MOI correlated with mIκBα protein expression in each of the cell lines. Importantly, transduction of cells with Ad-GFP at the same MOI had no effect on NF-κB transcriptional activity (data not shown). These results demonstrate effective inhibition of constitutive NF-κB activity in our panel of 5 thyroid cancer cell lines.

### The Role of NF-κB in Thyroid Cancer Cell Growth

**Figure 3:**
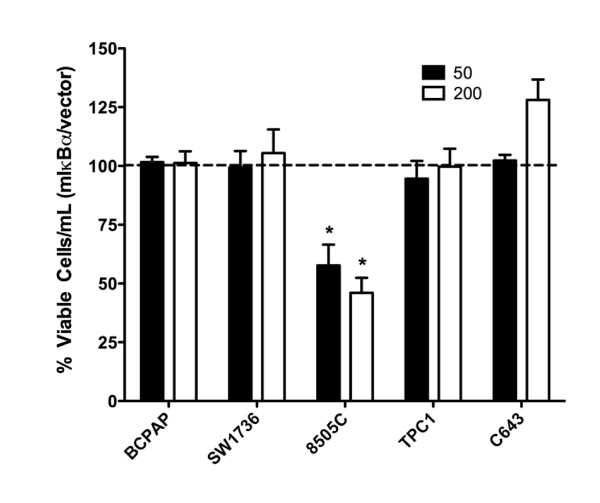
**Mutant IκBα Expression Inhibits Growth of the 8505C Anaplastic Thyroid Cancer Cell Line**. BCPAP, SW1736, 8505C, TPC1, and C643 cells were transduced with either Ad-GFP (as an appropriate control) or Ad-mIκBα at the indicated MOI. Cells were harvested for automated viable cell counting after 5 days. Data is represented as percent viable cells/mL compared to Ad-GFP control (normalized to 100%), which is denoted by the dashed line. Mean ± S.E.M. of 3 independent experiments is reported. [p < 0.05 (*); p < 0.01 (**); p < 0.001 (***)].

We next investigated the role of NF-κB in thyroid cancer cell proliferation and survival. Thyroid cancer cells were transduced with either Ad-mIκBα or Ad-GFP at an MOI of 50 or 200, and growth was assessed after 5 days by automated viable cell counting. Transduction with control Ad-GFP was performed to monitor transduction efficiency and control for the effects of adenoviral transduction on cell growth. Inhibition of NF-κB by mIκBα expression did not decrease thyroid cancer cell proliferation or survival in four of the five cell lines tested (Figure 3), even under conditions of serum starvation (0.5% FBS) (data not shown). However, the 8505C cell line showed a 42% decrease in cell growth in response to NF-κB inhibition when transduced with an MOI of 50. 8505C cell growth was inhibited with an MOI as low as 5 (36% inhibition; data not shown), confirming that NF-κB-dependent regulation of 8505C cell growth was not due to higher levels of mIκBα expression (Figure 2A).

**Figure 4:**
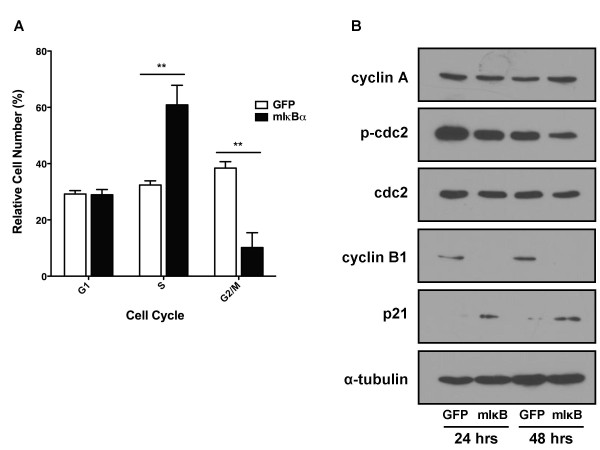
**Transition from S-phase to G2/M is Blocked in 8505C Cells in Response to NF-κB Inhibition**. **(A) **8505C cells were transduced with either Ad-GFP vs. Ad-mIκBα at an MOI of 25. After 48 hours, cells were harvested, and permeabilized. DNA was stained with a saponin/propidium iodide solution and subjected to cell cycle analysis by flow cytometry. Data is represented as relative cell number (%) in G1, S, or G2/M. Mean ± S.E.M. of 2 independent experiments performed in duplicate is reported. [p < 0.05 (*); p < 0.01 (**); p < 0.001 (***)] **(B) **8505C cells were transduced with either Ad-GFP or Ad-mIκBα at an MOI of 25. Whole-cell lysates were prepared 24 and 48 hours post-transduction, and levels of cyclin A, cdc2, phospho-cdc2, cyclin B1, and p21 were assessed by Western blot analysis. α-tubulin was used as a loading control.

To determine the mechanisms governing growth inhibition by mIκBα in the 8505C cell line, measures of apoptosis and cell cycle analysis were performed. Cleaved poly (ADP-ribose) polymerase (PARP), a measure of apoptosis, was undetectable by Western blot analysis, suggesting that NF-κB inhibition does not induce apoptosis (data not shown). However, flow cytometry revealed a significant increase in the number of cells in S-phase following mIκBα expression. This finding corresponded with a 28% decrease in the number of mIκBα-expressing cells in G2/M, indicating a block in the S-phase to G2/M transition (Figure 4A). Western blot analysis (Figure 4B) of cell cycle regulatory proteins demonstrated no regulation of cyclin A protein levels and only a small decrease in phospho-cdc2 levels in response to NF-κB inhibition. Notably, NF-κB inhibition resulted in an increase in p21 levels and decrease in the levels of cyclin B1, suggesting that the transition from S-phase to G2/M in 8505C cells is mediated by NF-κB-dependent regulation of cyclin B1 and p21. Based on the profound growth arrest observed in 8505C cells by NF-κB inhibition, this cell line was not used in subsequent studies of TNFα-induced apoptosis or invasion.

### The Role of NF-κB in Resistance to TNFα-induced Apoptosis

**Figure 5:**
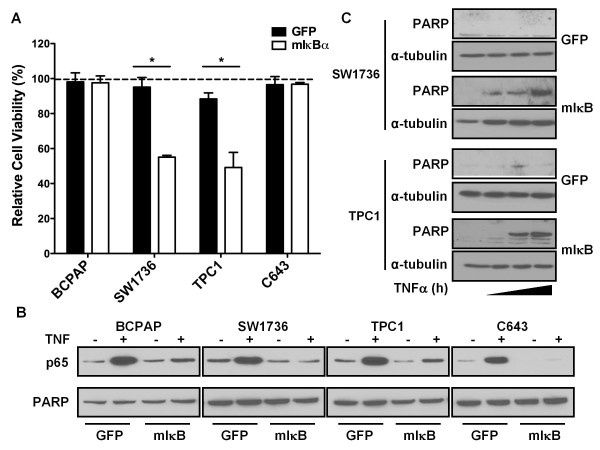
**NF-κB Inhibition Differentially Sensitizes Thyroid Cancer Cell Lines to TNFα-induced Apoptosis**. **(A) **BCPAP, SW1736, TPC1, and C643 cells were transduced with either Ad-GFP or Ad-mIκBα at an MOI of 200, 100, 50, and 100, respectively. After 24 hours, cells were treated with TNFα (10 ng/ml) or vehicle for 3 days. Cell viability was then assessed by MTS assay. Data is reported as relative cell viability (%) compared to vehicle-treated cells (normalized to 100%), which is denoted by the dashed line. Mean ± S.E.M. of 3 independent experiments performed in octuplicate is reported. [p < 0.05 (*); p < 0.01 (**); p < 0.001 (***)] **(B) **BCPAP, SW1736, TPC1, and C643 cells were transduced with either Ad-GFP or Ad-mIκBα at an MOI of 200, 100, 50, and 100, respectively. After 24 hours, cells were treated with TNFα (10 ng/ml) for 15 min. Nuclear fractions were prepared, and nuclear levels of p65 were analyzed by Western blot analysis. PARP was used as a nuclear loading control. **(C) **SW1736 and TPC1 cells were transduced with either Ad-GFP or Ad-mIκBα at an MOI of 100 and 50, respectively. After 24 hours, the cells were serum-starved overnight and then treated with 10 ng/ml TNFα for 0, 4, 8, and 16 hours. Whole-cell extracts were harvested, and Western blot analysis was performed to assess levels of cleaved PARP (denoted as PARP) as a marker of apoptosis.

TNFα signaling is responsible for activation of numerous pro-apoptotic pathways, which can be opposed by pro-survival NF-κB signaling through activation of the IKK complex [26]. Figure 5A (GFP) shows that thyroid cancer cell lines are resistant to TNFα-induced apoptosis, as cells transduced with control Ad-GFP displayed no significant decrease in cell viability following treatment with TNFα. We also observed that TNFα treatment resulted in the nuclear accumulation of p65, suggesting that increased NF-κB signaling promotes resistance to TNFα-induced apoptosis (Figure 5B; GFP). We therefore predicted that inhibition of TNFα-induced nuclear translocation of p65 would sensitize thyroid cancer cell lines to TNFα-induced apoptosis. As expected, expression of mIκBα greatly reduced nuclear translocation of p65 due to TNFα treatment, and lower levels of basal nuclear p65 were also observed in SW1736, TPC1, and C643 cells transduced with Ad-mIκBα when compared to control (Figure 5B). Interestingly, TNFα treatment in combination with NF-κB inhibition decreased cell viability in only two of the cancer cell lines (SW1736 and TPC1) (Figure 5A). Accordingly, levels of cleaved PARP, a marker of apoptosis, were increased after treatment with TNFα in SW1736 and TPC1 cells expressing mIκBα but not in cells expressing control GFP (Figure 5C). Cleaved PARP was not detected in TNFα/mIκBα resistant BCPAP or C643 cells (data not shown).

**Figure 6:**
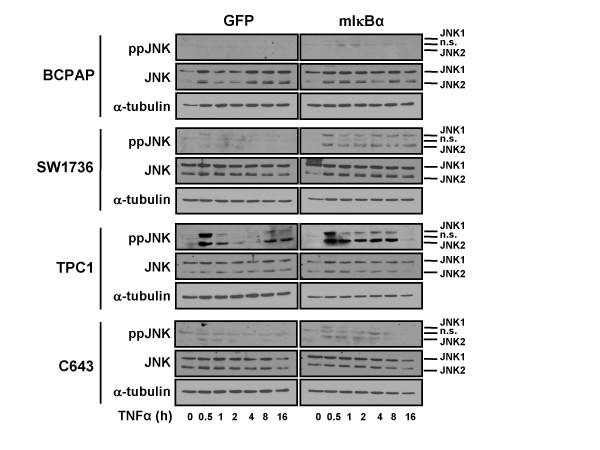
**Sustained Activation of the JNK Pathway Predicts Sensitivity to the Combinatorial Effects of Genetic NF-κB Inhibition and Treatment with TNFα**. BCPAP, SW1736, TPC1, and C643 cells were transduced with either Ad-GFP or Ad-mIκBα at an MOI of 200, 100, 50, and 100, respectively. Whole-cell extracts were harvested after 0.5, 1, 2, 4, 8 and 16 hours. Western blot analysis was performed to assess levels of ppJNK 1/2 (Thr-183/Tyr-185) and JNK 1/2. α-tubulin was used as a loading control. n.s. denotes a non-specific band (more prominent in BCPAP and C643 cells).

To investigate the mechanisms by which only a subset of cell lines are sensitized to the combined pro-apoptotic effects of TNFα treatment and NF-κB inhibition, we examined activation of the pro-apoptotic JNK/SAPK pathway. Activation of the JNK pathway was assessed by Western blot analysis with an antibody specific to the activated, phosphorylated (Thr183/Tyr185) forms of JNK1/2. Transient activation of JNK/SAPK pathway in control GFP-transduced cells was only observed in the TPC1 cell line (Figure 6). Interestingly, sustained activation of the JNK pathway was observed in response to TNFα treatment only in mIκBα-expressing cell lines (SW1736, TPC1) that were sensitive to the pro-apoptotic effects of combined TNFα treatment and genetic inhibition of NF-κB signaling (Figure 5A and 6).

### The Role of NF-κB in Thyroid Cancer Cell Invasion

**Figure 7:**
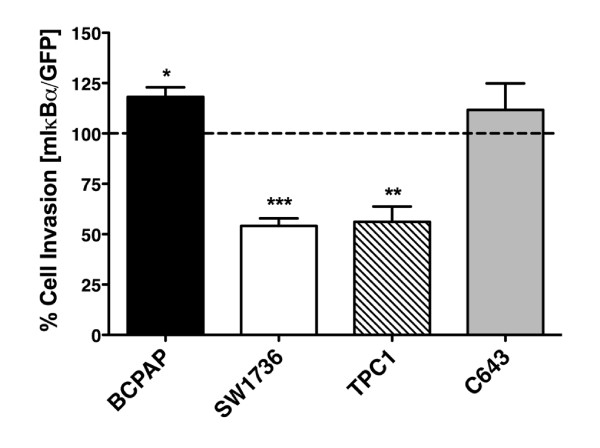
**Differential Inhibition of Thyroid Cancer Cell Line Invasiveness by Blocking NF-κB Signaling**. BCPAP, SW1736, TPC1, and C643 cells were transduced with either Ad-GFP or Ad-mIκBα at an MOI of 200, 100, 50, and 100, respectively. After 24 hours, cells were serum-starved in RPMI (0.1% FBS) for 6 hours. Cells were then harvested by trypsinization and applied to Matrigel-coated transwell chambers in RPMI supplemented with 0.1% FBS. Invasion was promoted by the presence of RPMI supplemented with 10% FBS in the lower chamber. Invaded cells were then fixed, stained with DAPI, and counted, as described in Materials and Methods. Data is represented as percent cell invasion compared to Ad-GFP control (normalized to 100%), which is denoted by the dashed line. Mean ± S.E.M. of 3 independent experiments performed in duplicate is reported. [p < 0.05 (*); p < 0.01 (**); p < 0.001 (***)].

An understanding of the role of NF-κB signaling in regulation of thyroid cancer cell invasion is particularly relevant given the nature of the disease and the mortality associated with locally invasive and metastatic tumors. Therefore, we assessed the role of NF-κB in thyroid cancer cell invasion using Matrigel-coated transwell assays. In these studies, cells (BCPAP, SW1736, TPC1, C643) were transduced with either Ad-GFP or Ad-mIκBα and allowed to invade for 24 hours. Control-transduced C643 cells were the most invasive (~450 cells/field), while TPC1 cells were the least invasive (~150 cells/field). SW1736 and BCPAP cells were moderately invasive (~300 cells/field) (data not shown). Invasion by the SW1736 and TPC1 cell lines was significantly inhibited (46% and 44%, respectively) by mIκBα expression, while BCPAP and C643 cells were resistant (Figure 7).

**Figure 8:**
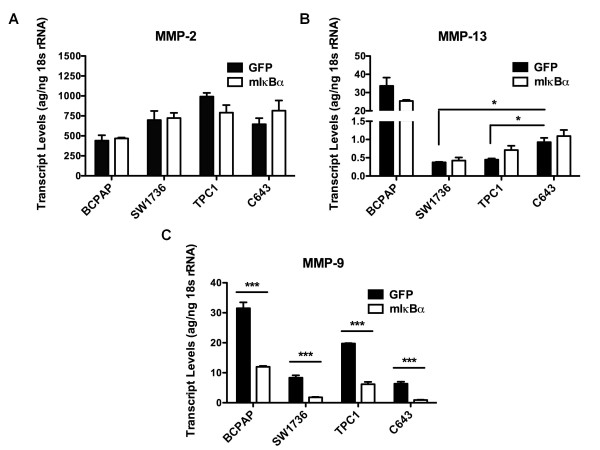
**Regulation of MMP-2, -9, and -13 Expression by NF-κB**. Quantitative RT-PCR was performed to analyze the regulation of MMP-2 **(a)**, -13 **(b)**, and -9 **(c) **transcript levels in cells in response to mIκBα expression. BCPAP, SW1736, TPC1, and C643 cells were transduced with either Ad-GFP or Ad-mIκBα at an MOI of 200, 100, 50, and 100, respectively. After 48 hours, total RNA was harvested. Data is represented as picograms target transcript corrected for nanograms 18s rRNA (internal standard) (MMP-2) or attagrams target transcript corrected for nanograms 18s rRNA (MMP-9, MMP-13). Mean ± S.E.M. of 3 independent experiments performed in duplicate is reported. [p < 0.05 (*); p < 0.01 (**); p < 0.001 (***)].

To investigate the mechanism by which NF-κB regulates thyroid cancer cell invasion, we performed quantitative RT-PCR to examine the NF-κB-dependent regulation of the matrix metalloproteinase (MMP) -2, MMP-9, and MMP-13 (Figure 8). Figure 8 shows that transcript levels of MMP-2 and MMP-13 were not significantly affected by mIκBα expression after 48 hours. Interestingly, both resistant cell lines (BCPAP, C643) expressed basal MMP-13 transcripts levels that were at least two-fold higher (p<0.05) than either of the sensitive cell lines, while MMP-2 levels were similar across all cell lines (Figure 8A, B). Figure 8C shows that MMP-9 transcript levels were decreased significantly by NF-κB inhibition in both the resistant [BCPAP (~2.5 fold), C643 (~7 fold)] and sensitive [SW1736 (~4.5 fold), TPC1 (~3 fold)] cell lines.

## Discussion

In this report, we have used a selective genetic inhibitor of NF-κB signaling to determine the effects of this pathway on proliferation, apoptosis, and invasion in a panel of ATC and PTC thyroid cancer cell lines. Our data indicate that NF-κB does not have one common role in the regulation of proliferation, apoptosis, or invasion in advanced thyroid cancer and that sensitivity to NF-κB inhibition does not correlate with baseline levels of NF-κB transcriptional activity. Instead, we have demonstrated that some cancer cells depend on NF-κB signaling for proliferation (8505C), while others require it for invasion and resistance to TNFα-induced apoptosis (TPC1, SW1736). Furthermore, some cell lines are not dependent on NF-κB signaling for these basic cancer properties (BCPAP, C643).

The hallmarks of cancer include self-sufficiency in growth signals, resistance to anti-growth signals, evasion of apoptosis, unlimited replication potential, sustained angiogenesis, and acquisition of metastatic/invasive potential [27]. Activation of NF-κB signaling in cancer is a critical mediator in the majority, if not all, of these processes [11]. This theory has led to numerous studies aimed at identifying a link between NF-κB signaling and thyroid cancer growth and progression. Using the NF-κB inhibitor DHMEQ, Yamashita and colleagues showed decreased tumor growth, cancer cell invasion, increased apoptosis, as well as TNFα- and taxane-induced apoptosis in a single ATC cell line [13,14]. Liu and Xing demonstrated synergistic inhibition of cell proliferation in a panel of thyroid cancer cell lines when combining a MEK1/2 inhibitor with PS1145, an IKK complex inhibitor [17]. Another study by Zhu and colleagues showed that a small molecule triptolide inhibited angiogenesis, invasion, and proliferation in a single ATC cell line and further suggested that this was associated with inhibition of NF-κB transcriptional activity [15]. The primary genetic study linking NF-κB to thyroid cancer was performed by Pacifico and colleagues via stable overexpression of mIκBα in the FRO ATC cell line. In doing so, they showed that NF-κB promotes *in vivo *tumor formation, growth in soft-agar, and resistance to chemotherapy-induced apoptosis [28]. Our studies have extended the use of a genetic model to identify a role for NF-κB signaling in a panel of authenticated thyroid cancer cell lines, and in doing so, we have advanced knowledge of the role of NF-κB in thyroid cancer proliferation, apoptosis, and invasion.

Previous studies in cancer models, including thyroid cancer, have cited a role for NF-κB in malignant cell proliferation by transcriptional regulation of cyclin D1, CDK2, cyclin E and c-Myc [11]. Using a selective genetic model, however, we have shown that NF-κB regulates cancer cell proliferation in only one of five cell lines tested (Figure 3). Cell cycle arrest was induced in the 8505C ATC cell line by blocking the transition from S-phase to G2/M (Figure 4A), representing a novel mechanism for the regulation of thyroid cancer cell proliferation by NF-κB. While cyclin A is critical for progression through S-phase [29], protein levels were not affected by NF-κB inhibition (Figure 4B). Similarly, dephosphorylation of cdc2 at Tyr 15, which is predicted upon entry into G2/M [29], was observed (Figure 4B). However, protein levels of cyclin B1, which is necessary for the entry into and progression through G2-phase and mitosis [29], were decreased following NF-κB inhibition (Figure 4B). Interestingly, this finding was associated with increased levels of the cyclin-dependent kinase inhibitor, p21 (Figure 4B). Indeed, increased levels of p21 have been shown to mediate p53-induced cell cycle arrest in response to genotoxic stress by down-regulation of cyclin B1 expression [30]. NF-κB signaling has also been shown to inhibit cell cycle arrest by decreasing p21 levels in osteoblasts [31] and normal epithelial cells in a manner dependent on the phosphatidylinositol-3-kinase (PI3K) pathway [32]. Similar observations have been made in prostate cancer cells [33], thereby providing substantial evidence for a mechanism by which decreased NF-κB signaling in the 8505C cell line leads to decreased cyclin B1 expression in a p21-dependent manner.

The ability of NF-κB signaling to deregulate programmed cell death by apoptosis is a major mechanism by which NF-κB exerts its pro-tumorigenic functions. This effect is often achieved by transcriptional regulation of anti-apoptotic genes, including Bcl-2 family members [11,13,34]. Starenki and colleagues demonstrated that inhibition of NF-κB by DHMEQ in thyroid cancer cells induced spontaneous apoptosis through down-regulation of cIAP-1, cIAP-2, and XIAP [13]. However, our studies indicate that genetic inhibition of NF-κB in a panel of thyroid cancer cell lines does not induce spontaneous apoptosis even under conditions of serum-starvation (Figure 3 and data not shown). Pacifico and colleagues obtained similar results when stably overexpressing mIκBα in the FRO ATC cell line [28].

NF-κB signaling is critical for blocking apoptosis following ligand binding by members of the tumor necrosis factor receptor (TNFR) superfamily. Members of this family induce activation of the extrinsic apoptosis pathway by a cytoplasmic death domain. These receptors include the classical receptor TNFR1, which binds the inflammatory cytokine TNFα, as well as Fas, TNF-related apoptosis-inducing ligand (TRAIL)-receptor 1, and TRAIL-R2 [35,36]. Pro-apoptotic effects of TNFR1 activation by TNFα are two-fold. First, receptor activation induces formation of complex II, which ultimately triggers mitochondrial release of cytochrome c, and second, activation of the JNK/SAPK pathway results in downstream release of Smac/Diablo into the cytosol to activate caspase 8. However, under conditions in which NF-κB signaling is intact, the pro-apoptotic functions of TNFR1 activation are often masked due, in large part, to the survival functions imparted by NF-κB. Indeed, studies have shown that abrogation of NF-κB signaling can enhance TNFα-induced apoptosis [36]. Our results demonstrate that inhibition of NF-κB signaling promotes TNFα-induced apoptosis in a subset of cell lines, and this finding is associated with a sustained activation of JNK in response to TNFα-treatment in the presence NF-κB inhibition in these cell lines (SW1736, TPC1) (Figure 6). Sustained activation of JNK was not observed in the resistant cell lines (BCPAP, C643). These data suggest a mechanism by which sensitization of thyroid cancer cell lines to the combinatorial effects of TNFα-treatment and NF-κB inhibition requires activation of the JNK pathway that may, in fact, be cell line and tumor specific. However, the exact mechanism by which differential activation of JNK remains unclear and may involve variable regulation of upstream signaling components. One study showed that breast cancer cells, which are typically resistant to TNFα-induced apoptosis, demonstrated enhanced apoptosis and prolonged activation of JNK when expressing mIκBα [37]. The authors were able to block induction of apoptosis via treatment with an inhibitor of JNK activation. In our studies, however, treatment with the JNK inhibitor, SP600125, alone inhibited growth of our thyroid cancer cell lines (data not shown), likely due to off-target mechanisms associated with the compound, as described by Bain and colleagues [38]. Another study reported a cell line-specific, differential activation of JNK following treatment of two oral squamous cell carcinoma (OSCC) lines with TRAIL, another ligand known to activate the TNFR superfamily [39]. While this study did not involve inhibition of NF-κB, their basic findings were consistent with ours, in that a strong induction of JNK activation was necessary for OSCC sensitivity to TRAIL-induced apoptosis.

MMPs belong to a family of endopeptidases, which are classified based on their specificity for particular extracellular matrix substrates, and are believed to play a critical role in the acquisition of metastatic potential by cancer cells by promoting migratory/invasive potential. MMP regulation is governed by numerous oncogenic processes, including constitutive activation of NF-κB [40,41]. In the current study, we have demonstrated differential regulation of invasion by NF-κB and evaluated the expression levels of MMP-2, -9, and -13. These MMPs are significant in that they are regulated by NF-κB (MMP-9) and expressed ubiquitously in thyroid cancer cell lines (MMP-13) [41,42]. Also, expression of both MMP-2 and MMP-9 is increased in neoplastic thyroid cell lines when compared to normal thyroid cell lines [41,42]. Only MMP-9 displayed significantly decreased transcript levels in response to NF-κB inhibition (Figure 8C). This finding is important, however, given the correlation with MMP-9 expression and poor prognosis in breast and prostate cancer. No significant regulation of MMP-2 or MMP-13 was observed. Interestingly, MMP-13 transcript levels at baseline were at least two-fold higher in the resistant cell lines (BCPAP, C643) when compared to transcript levels in sensitive cell lines (SW1736, TPC1) (Figure 7; Figure 8B). Further studies will be required to determine the precise mechanisms by which NF-κB regulates invasion in thyroid cancer cells. Nonetheless, the insights provided in this study clearly demonstrate a role for NF-κB in thyroid cancer cell invasion.

## Conclusions

In conclusion, our results demonstrate an important and diverse role for NF-κB signaling in thyroid cancer. Interestingly, these effects are not observed across an entire panel of thyroid cancer cell lines, and they are not associated with a particular mutational status or histological tumor classification. Here, we show distinct roles for NF-κB signaling in the regulation of thyroid cancer cell proliferation, resistance to TNFα-induced apoptosis, and invasion. Decreased proliferation through blockade of the S-phase to G2/M transition is observed in response to NF-κB inhibition. Furthermore, NF-κB likely mediates cancer cell invasion, at least in part, by driving MMP-9 transcription. Finally, sensitivity to TNFα-induced apoptosis by inhibition of NF-κB is associated with sustained activation of the JNK pathway. Taken together, these results suggest that novel therapeutics targeting NF-κB may be of clinical utility in the treatment of advanced thyroid cancer, but this is not likely to be of global use in the treatment of all thyroid cancers. Downstream markers may identify which cell lines, and ultimately which patients, may respond to inhibitors of this important pathway.

## Methods

### Cell Culture

The BCPAP, SW1736, 8505C, TPC1, and C643 thyroid cancer cell lines were maintained for 10-20 passages at 37°C and 5% CO_2 _in RPMI 1640 (Invitrogen) supplemented with 10% fetal bovine serum (FBS) (Hyclone). The BCPAP and 8505C cell lines were kindly provided by Dr. M. Santoro (Medical School, University "Federico II" of Naples, Naples, Italy). The SW1736 and C643 cell lines were kindly provided by Dr. K. Ain (University of Kentucky), with permission from Dr. N-E Heldin (University Hospital, S-751 85 Uppsala, Sweden). The TPC1 cell line was kindly provided by Dr. S. Jhiang (Ohio State University). Cell lines were routinely profiled by Short Tandem Repeat analysis and are consistent with our previously published profiles [18].

### NF-κB Inhibitors

The NF-κB inhibitors, IKK Inhibitor VII (Calbiochem), Bay 11-7082 (Calbiochem), and CDDO-Me (kindly provided by Dr. M. Sporn, Dartmouth Medical School, Hanover, NH), were dissolved in DMSO at a final concentration of 10 mM.

### Adenoviral Transductions

Expression of mIκBα was achieved by adenoviral transduction. Ad-mIκBα and Ad-GFP were kindly provided by Dr. J. DeGregori (University of Colorado Denver, Anschutz Medical Campus, Aurora, CO) [25]. For transduction, cells were trypsinized and resuspended in RPMI supplemented with 1% FBS, transduced in suspension for 1 hour with gentle agitation, and plated at the indicated cell number in RPMI supplemented with 10% FBS.

### Transfections and Reporter Assays

A 3×-κB NF-κB-responsive luciferase reporter comprised of 3 tandem repeats from the MHC class I enhancer and a β-galactosidase reporter driven by the actin promoter (actin β-gal) were kindly provided by Drs. A. Baldwin (University of North Carolina) and M. Karin (University of California, San Francisco), respectively. Transfections were carried out using Lipofectamine 2000 (Invitrogen), according to manufacturer's protocol. Briefly, thyroid cancer cells (1.0 × 10^5^) were transduced with either Ad-GFP (as an appropriate control) or Ad-mIκBα at the indicated MOI and seeded in 24-well plates 24 hours prior to transfection. DNA (0.8 μg) and Lipofectamine were diluted in Optimem at a Lipofectamine:DNA ratio of 2.5:1. After 4-6 hours, the media containing the complexes was removed from cells and replaced with fresh RPMI supplemented 10% FBS. Cells were harvested at 24 hours post-transfection in passive lysis buffer (PLB; Promega) and subjected to a single freeze-thaw cycle. The Luciferase Assay System (Promega) and Luminescent β-galactosidase Detection Kit II (Clontech) were used to assay luciferase and luminescent β-galactosidase activities, respectively. Luciferase relative light units (RLU) were normalized to luminescent β-galactosidase activity (RLU) to obtain a normalized measure of NF-κB transcriptional activity.

### Western Blot Analysis

To prepare whole-cell extracts, cells were washed once with phosphate-buffered saline (PBS) and harvested in extraction buffer (EB; 1% Triton X-100, 10 mM Tris, pH 7.4, 5 mM EDTA, 50 mM NaCl, 50 mM NaF, 1 mM phenylmethylsulfonyl fluoride, and 2 mM Na_3_VO_4 _supplemented with 1× complete protease inhibitors (Roche Diagnostics). Cellular debris was then removed by centrifugation at 13,000 rpm for 10 minutes at 4°C. Nuclear and cytoplasmic extracts were prepared using the Nuclear Extract Kit (Active Motif), according to manufacturer's instructions. Extracts (40 μg) were resolved on 10% SDS-PAGE and transferred to Immobilon-P transfer membranes (Millipore). Membranes were probed with primary antibodies against PARP (rabbit polyclonal, AB16661, Millipore), cleaved PARP (mouse monoclonal, AB3565, Millipore), α-tubulin (mouse monoclonal, DM1A, Calbiochem), RelA (mouse monoclonal, F-6, Santa Cruz), IκBα (rabbit polyclonal, FL, Santa Cruz), ppSAPK/JNK (mouse monoclonal, #9255s, Thr-183/Tyr-185, Cell Signaling), SAPK/JNK (rabbit polyclonal, #9252, Cell Signaling), cyclin B1 (rabbit polyclonal, #4138, Cell Signaling), cyclin A (mouse monoclonal, #4656, Cell Signaling), and p21 (rabbit monoclonal, #2947, Cell Signaling) in blocking buffer (5% nonfat dry milk in 20 mM Tris, pH 7.4, 138 mM NaCl, 0.1% Tween (TBST)) overnight at 4°C. Membranes were washed with TBST and incubated with secondary goat anti-rabbit or goat anti-mouse horseradish peroxidase-conjugated antibody (GE Healthcare) for 3 h at room temperature. Immunoreactivity was visualized by enhanced chemiluminescence detection (Pierce). Blots were stripped and re-probed, where indicated, using Re-Blot Plus Mild (Millipore).

### Automated Cell Counting Assays

For studies with pharmacologic inhibitors of NF-κB, thyroid cancer cells (1.5 × 10^4 ^- BCPAP, C643, SW1736, and 8505C; 7.5 × 10^3 ^- TPC1) were seeded in 6 cm plates. The following day, cells were treated with either vehicle (DMSO), IKK Inhibitor VII (1 μM), Bay 11-7082 (1 μM), or CDDO-Me (0.25 μM). Cells were replenished with fresh RPMI (10% FBS) supplemented with drug or vehicle after two days. After a total of five days of treatment, the media was collected, and adherent cells were washed with PBS and harvested by trypsinization. Cells were then combined with the collected media, centrifuged at 1,000 rpm for 5 minutes, and resuspended in 0.5 ml PBS. Viable cells were then counted using the Vi-CELL Coulter Counter (Beckman, Inc). For viral transduction studies, cells (1.5 × 10^4 ^- BCPAP, C643, SW1736, and 8505C; 7.5 × 10^3 ^- TPC1) were transduced as described above with either Ad-GFP or Ad-mIκBα at an MOI of 50 or 200 and then seeded in 6 cm plates. On the following day, the media was replaced with fresh RPMI (10% FBS). The media was again replaced with fresh RPMI (10% FBS) two days later. Five days post-transduction, media and cells were collected, and viable cell number was assessed as described above by ViCell counting.

### Cell Cycle Analysis

8505C thyroid cancer cells (7.5 × 10^5^) were transduced as described above with either Ad-GFP or Ad-mIκBα at an MOI of 25. Cells were then seeded in 10 cm dishes in RPMI (10% FBS). After 48 hours, adherent cells were harvested by trypsinization and washed with PBS. Cell pellets were resuspended in a saponin/propidium iodide solution (0.3% saponin, 25 μg/mL propidium iodide, 0.1 mM EDTA, and 10 μg/mL RNase A). Cells were incubated at 4°C for 8 hours, and cell cycle distribution was determined by flow cytometry using a Beckman Coulter FC500 at the University of Colorado Cancer Center Flow Cytometry Core. ModFit LT (Verity Software House) was used for cell cycle modeling and doublet discrimination.

### Cell Viability Assays

Thyroid cancer cells were transduced with either Ad-GFP (as an appropriate control) or Ad-mIκBα at an MOI sufficient to achieve greater than 90% of NF-κB transcriptional activity, as determined by luciferase assay (BCPAP-200; SW1736-100; TPC1-50; C643-100). Cells (2,500/well for TPC1; 5,000/well for BCPAP, SW1736, and C643) were seeded in octuplicate into 96-well plates in RPMI supplemented with 10% FBS. Cells were treated the next day with medium containing 10 ng/ml TNFα (GenScript) (or vehicle) for three days, and cell viability was assayed after three days. Cell viability was measured per manufacturer's instructions using the CellTiter 96 Aqueous Non-Radioactive Cell Proliferation Assay (Promega) with an MRX Microplate Reader (Dynatech Laboratories) and the Revelation software at an absorbance of 490 nm.

### Assessment of Apoptosis

Apoptosis was assessed by Western blot analysis of cleaved PARP, as described above. For these studies, cells were serum-starved overnight prior to treatment. Cleaved PARP is the 89 kD cleavage product of poly (ADP-ribose) polymerase 1 (PARP1), which serves as a caspase substrate during the early stages of apoptosis [43].

### Invasion Assays

Thyroid cancer cells were transduced as described above with either Ad-GFP or Ad-mIκBα at an MOI sufficient to achieve greater than 90% inhibition of NF-κB transcriptional activity, as determined by luciferase assay (BCPAP-200; SW1736-100; TPC1-50; C643-100). Twenty-four hours later, the media was replaced with fresh RPMI (0.1% FBS). After 6 hours, cells (1.0 × 10^5^) were harvested and seeded in the upper chambers of Matrigel-coated transwell chambers (24-well, 8 μM pore size; BD Biosciences) in RPMI (0.1% FBS). Cell invasion was promoted by the presence of RPMI supplemented with 10% FBS in the lower chamber. After 24 hours, non-invading cells on the top chamber were removed by scraping with a cotton swab, and invading cells on the lower surface were fixed with methanol and stained with 3 μg/mL 4',6-diamidino-2-phenylindole (DAPI; Invitrogen). Cell invasion was assessed by counting DAPI-stained nuclei in five microscopic fields under 10× magnification using the Metamorph software (Molecular Devices) and Nikon microscope.

### Quantitative RT-PCR

Thyroid cancer cells were transduced with either Ad-GFP (as an appropriate control) or Ad-mIκBα at an MOI sufficient to achieve greater than 90% of NF-κB transcriptional activity, as determined by luciferase assay (BCPAP-200; SW1736-100; TPC1-50; C643-100). Total RNA was isolated from the cells after 48 hours using the PerfectPure RNA Cultured Cell Kit (5 Prime) as per the manufacturer's protocol. The mRNA levels of MMP-2, -9, and -13 were measured by real-time quantitative reverse transcription-PCR using ABI PRISM 7700.

*MMP-2*; TaqMan Gene Expression Assay ID: HS00234422_m1.

*MMP-9; *Sense-CCCGGACCAAGGATACAGTTT, Antisense- GAATGATCTAAGCCCAGCGC, Probe-CCTCGTGGCGGCGCATGAG

*MMP-13*; Sense-TGGCATTGCTGACATCATGA; Antisense-GCCAGAGGGCCCATCAA, Probe-TGGAATTAAGGAGCATGGCGACTTCTACC

Amplification reactions and thermal cycling conditions were optimized for each primer-probe set and carried out by the University of Colorado Cancer Center QRT-PCR Core. A standard curve was generated using the fluorescent data from the 10-fold serial dilutions of control RNA. This was then used to calculate the transcripts levels of MMP-2, -9, and -13 in the RNA samples. Quantities of MMP-2, -9, and -13 in samples were normalized to the corresponding 18S rRNA (PE ABI, P/N 4308310).

### Statistical Analysis

Statistical significance between groups was determined using the two-tailed t-test (GraphPad Prism) [p < 0.05 (*); p < 0.01 (**); p < 0.001 (***)]

## Competing interests

The authors declare that they have no competing interests.

## Authors' contributions

KTB designed and performed the experiments, interpreted data, assembled the figures, and drafted the manuscript. BRH conceived of the study and directed the research, with assistance from RES. RES and BRH critically reviewed the manuscript, which was approved by all authors.
